# Genome-Wide Identification and Expression Analysis of *ACA*/*ECA*s in *Capsicum annuum* L.

**DOI:** 10.3390/ijms252312822

**Published:** 2024-11-28

**Authors:** Yuxuan Qian, Jing Tong, Ning Liu, Baoju Wang, Zhanhui Wu

**Affiliations:** 1 Beijing Vegetable Research Center, Beijing Academy of Agriculture and Forestry Science, Beijing 100097, China; 2 Key Laboratory of Urban Agriculture (North) of Ministry of Agriculture and Rural Affairs, Beijing 100097, China; 3 National Key Laboratory of Biological Breeding, Beijing 100097, China; 4 National Vegetable Engineering Technology Research Center, Beijing 100097, China

**Keywords:** pepper, *ACA*/*ECA*, genome-wide identification, gene expression, calcium, abiotic stress

## Abstract

Pepper (*Capsicum annuum* L.) is a popular vegetable in people’s daily lives. During pepper growth, calcium (Ca) is an essential macronutrient, and calcium-transporting ATPase (ACA/ECA) is a vital protein for calcium transport. However, reports on the *ACA*/*ECA* gene family in the pepper genome are lacking. Hence, we used various bioinformatics methods to identify the *ACA*/*ECA* gene family in pepper. We identified eleven *CaACA*/*ECA*-family genes in pepper. The chromosomal distribution, phylogenetic evolution, characteristics, gene collinearity, gene and protein structures, cis-acting elements, and specific expression patterns of *CaACA*/*ECA*s were analyzed, revealing evolutionary relationships and correlations between *CaACA*/*ECA*s and other species (Arabidopsis, rice, and tomato). The experimental results indicate that *CaACA/ECAs* are stable and hydrophobic proteins, with each of the eleven *CaACA/ECA* proteins containing all ten motifs. Eleven *CaACA*/*ECA* genes are unevenly distributed on the eight chromosomes, and they substantially differ in the number of exons. We found a close correlation between the *ACA*/*ECA*s of pepper, Arabidopsis, and tomato. The *CaACA*/*ECA* genes contain various plant-hormone-, growth-, and stress-related cis-acting elements. The qRT-PCR results indicate that the expression levels of the eleven *CaACA*/*ECA*s exhibit differential temporal expression patterns under various exogenous Ca^2+^ concentrations. These results provide a theoretical basis for further studying the function of the pepper *ACA*/*ECA* gene family and valuable information for identifying and screening genes for pepper stress tolerance breeding.

## 1. Introduction

Pepper (*Capsicum annuum* L.) is a thermophilic and heliophilic vegetable native to South America and one of the main vegetables grown in greenhouses. Pepper is rich in various vitamins and minerals and is a common vegetable in people’s daily lives [1,2]. Calcium (Ca) is an essential macronutrient during pepper growth, playing a central regulatory role in its growth and adaptation to environmental stress [3]. Ca^2+^ is a conserved and versatile signaling modulator involved in the response to biotic and abiotic stress, immunity, nodulation, and circadian rhythms [4].

Cells activate receptor proteins with high affinity for Ca^2+^ under stress to maintain the cytoplasmic Ca^2+^ concentration in a state of equilibrium [5]. Calcium-transporting ATPase, a vital protein for calcium transport, plays a crucial role in regulating plant growth and maintaining the balance of intracellular calcium ions [6]. Ca^2+^-ATPase catalyzes the hydrolysis of ATP on the inner side of the plasma membrane, releasing energy and driving intracellular calcium ions to be pumped out of the cell or into the endoplasmic reticulum cavity for storage, thereby maintaining a low concentration of Ca^2+^ for homeostasis in the cell [7]. Calcium-transporting ATPases are classified into two subfamilies according to their amino acid sequences and biochemical features: endoplasmic reticulum calcium-transporting ATPase (ECA) and plasma membrane calcium-transporting ATPase (ACA) [8]. ECA is distributed among the endoplasmic reticulum, vacuolar membrane, and plasma membrane, whereas ACA is found in nearly all membrane systems within the cell [9,10]. The critical functions of the *ACA*/*ECA* genes, especially in abiotic stresses, are being uncovered as research progresses. The expression level of *AtACA4* increases in Arabidopsis when subjected to salt stress. Expressing *AtACA4* in yeast enhances its salt tolerance, indicating that AtACA4 plays a crucial role in Ca^2+^ signaling during salt stress [11]. The expression levels of *AtACA8* and *AtACA9* in Arabidopsis seedlings treated with abscisic acid gradually increase [12]. The transcription level of *OsACA6* increases in rice under salt stress or abscisic acid treatment, whereas the expression level of *OsACA5* markedly increases under salt stress [13,14]. The expression level of *GsACA1* is upregulated in wild soybean under salt and alkali stress [15]. Furthermore, ACA and ECA are sensitive to variations in Ca^2+^ concentration. For instance, the expression levels of the majority of *ACA* genes in bananas were high when subjected to 2 and 4 mM Ca^2+^ treatments, whereas their expressions were strongly suppressed under 0 mM Ca^2+^ and 1 mM Ca^2+^ treatments [16]. ACA and ECA affect plant growth. For example, the lack of *ECA* genes in Chinese cabbage leads to slow new leaf growth, as well as the withering and yellowing of leaves [17]. These findings indicate that the *ACA*/*ECA* gene family is important in various plants.

The *ACA*/*ECA* gene family has been identified and functionally analyzed in various plants. However, reports on the *ACA*/*ECA* gene family in the pepper genome are lacking, and studies on the functions of this gene family are limited. As such, we used various bioinformatics methods to identify the *ACA*/*ECA* gene family in pepper. Our objectives were to (1) identify the *ACA*/*ECA* gene family in the pepper genome, (2) analyze their expression dynamics under various exogenous Ca^2+^ concentrations, and (3) predict their responses and adaptations to abiotic stresses.

## 2. Results

### 2.1. Identification and Analysis of ACA/ECA Gene Family in Pepper

**Figure 1:**
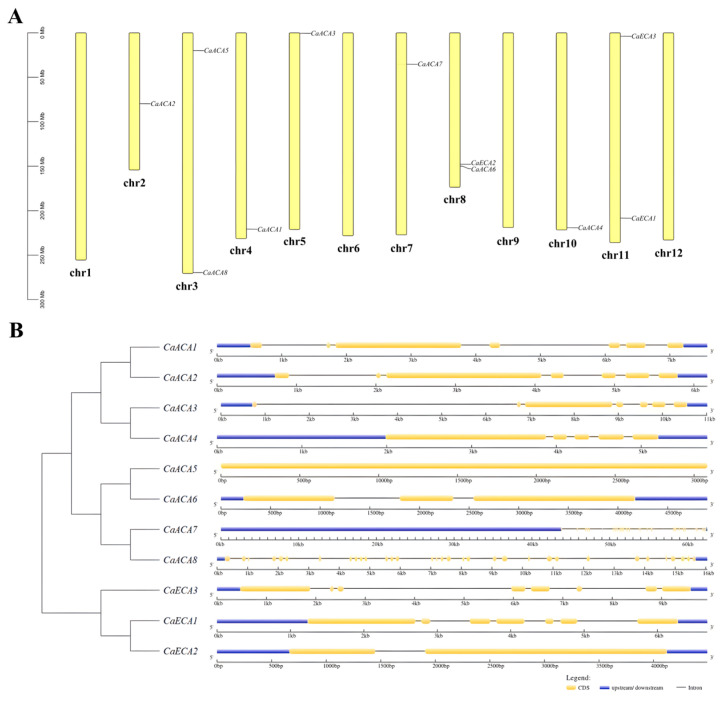
Chromosome distribution and structures of *CaACA*/*ECA* genes. (**A**) *CaACA*/*ECA* genes on pepper chromosomes. The yellow rectangular bars represent the pepper chromosomes, and the 0–300 Mb scale represents the chromosome length. The gene ID of *CaACA*/*ECA* is highlighted in black for the corresponding chromosome. (**B**) Exon–intron structures of *CaACA*/*ECA* genes.

Eleven members of the *ACA*/*ECA* gene family were identified in the pepper genome using BlastP analysis. The distribution of these eleven *ACA*/*ECA* genes is disproportionate across the eight pepper chromosomes. Chromosome 8 has one *ACA* and one *ECA* and chromosome 11 has two *ECA*s, whereas chromosome 1, 6, 9, and 12 are devoid of any members of this gene family (Figure 1A). We analyzed the structural composition of the *CaACA*/*ECA* genes to explore differences in the gene architecture, and we found that the number of exons in all *CaACA*/*ECA* genes varies widely, ranging from 1 to 33 (Figure 1B).

The results of the bioinformatics profiling of the eleven *ACA*/*ECA* genes show that the number of amino acids and molecular weights of CaACA/ECA range from 940 (CaACA4) to 1083 (CaACA7) and 103,249.83 (CaACA4) to 117,887.24 (CaACA8), respectively. The theoretical isoelectric points range from 5.23 (CaECA1) to 8.96 (CaACA5), with an average value of 6.54, which includes eight acidic proteins (pI < 7) and three alkaline proteins (pI > 7). The instability coefficient ranges from 33.07 (CaACA6) to 39.56 (CaECA1), with an average of 36.74 (<40), indicating that CaACA/ECAs are stable proteins. The hydrophilic results indicate that CaACA/ECA proteins are hydrophobic (>0). In addition, the subcellular prediction results of the CaACA/ECA proteins align with the subcellular localization characteristics of its protein family (Table 1).

### 2.2. Protein Phylogenetic and Gene Collinearity Analyses of ACA/ECA Family from Arabidopsis, Rice, Tomato, and Pepper

**Figure 2:**
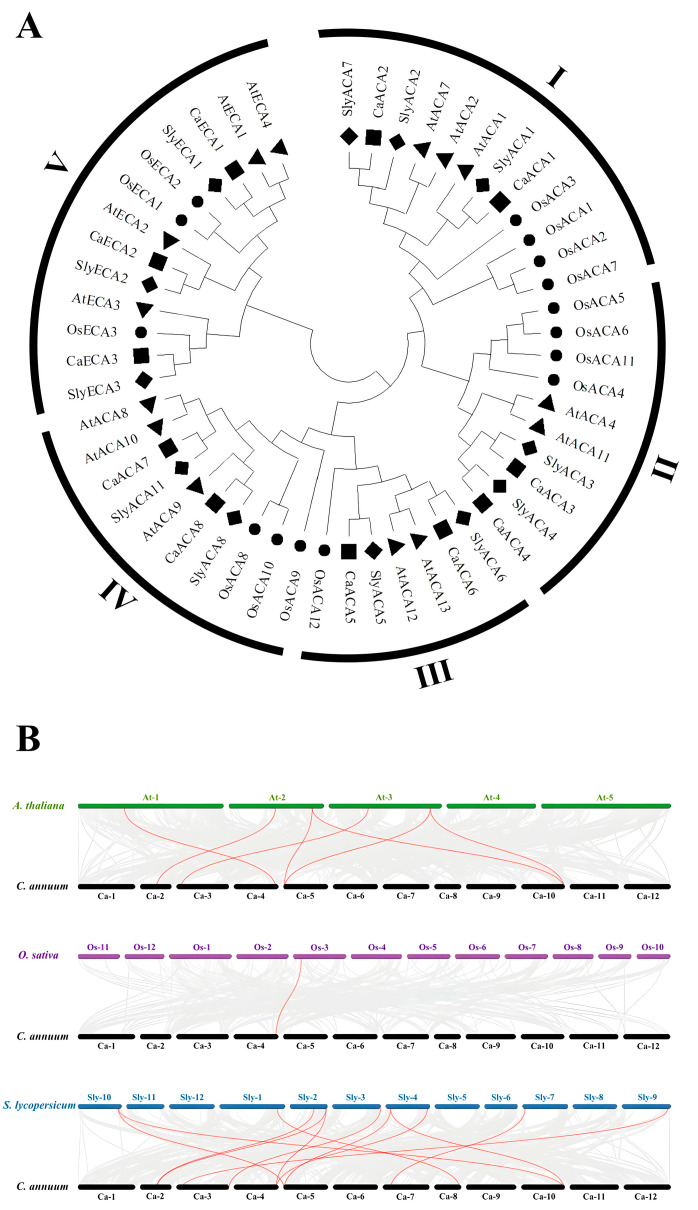
The phylogenetic tree of ACA/ECA proteins and the collinear analyses of *ACA*/*ECA* genes across plant species. (**A**) The phylogenetic tree of ACA/ECA proteins from *Arabidopsis thaliana*, *Oryza sativa*, *Solanum lycopersicum,* and *Capsicum annuum* were constructed using MEGA 11.0.13 software with 1000 bootstrap replications. The triangles represent *Arabidopsis thaliana*, the circles represent *Oryza sativa*, the rhombuses represent *Solanum lycopersicum*, and the squares represent *Capsicum annuum*. (**B**) The results of the collinearity analyses of pepper *ACA*/*ECA* genes with Arabidopsis, rice, and tomato. The chromosomes are distinctly colored. The gray lines in the background indicate the collinear blocks within pepper and other plant genomes. The red lines connect syntenic *ACA*/*ECA* gene pairs.

Multiple sequence alignments were conducted and the phylogenetic trees of the ACA/ECA proteins isolated from four species were constructed to evaluate the evolutionary development of the *ACA*/*ECA* gene family. The ACA/ECA protein family is categorized into five highly conserved evolutionary branches (I–V), comprising twelve, ten, seven, ten, and thirteen ACA/ECA proteins, respectively. Within this classification, CaACA/ECA proteins are distributed across all five clades (Figure 2A).

The collinearity of pepper with Arabidopsis, rice, and tomato was analyzed to understand the phylogenetic affiliations among the *ACA*/*ECA* genes of pepper and other plant species. The results of our interspecific collinearity analyses revealed that *CaACA*/*ECA* exhibits seven collinear gene pairs with Arabidopsis, one collinear gene pair with rice, and twelve collinear gene pairs with tomato (Figure 2B and Table S1). These results reveal that the genetic relationship of *ACA*/*ECA* genes between pepper and dicotyledonous plants is closer than that with monocotyledonous plants, with tomato being the most closely related.

### 2.3. Conserved Motif Analysis of the CaACA/ECA-Family Proteins

**Figure 3:**
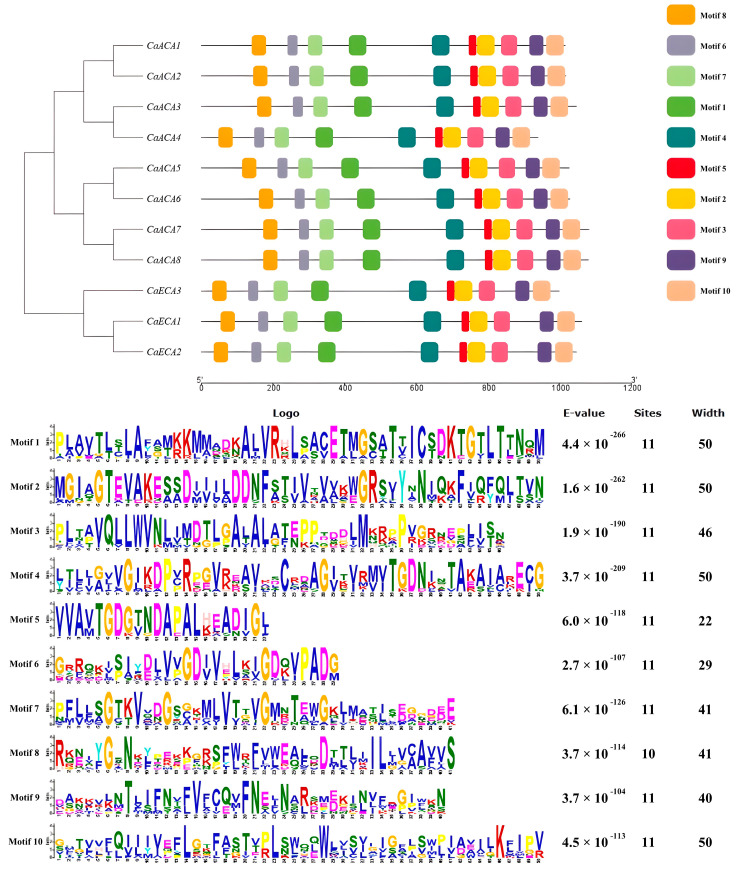
Conserved motifs and sequences of CaACA/ECAs. Ten conserved motifs of CaACA/ECAs were identified using MEME and are indicated with different colors.

**Figure 4:**
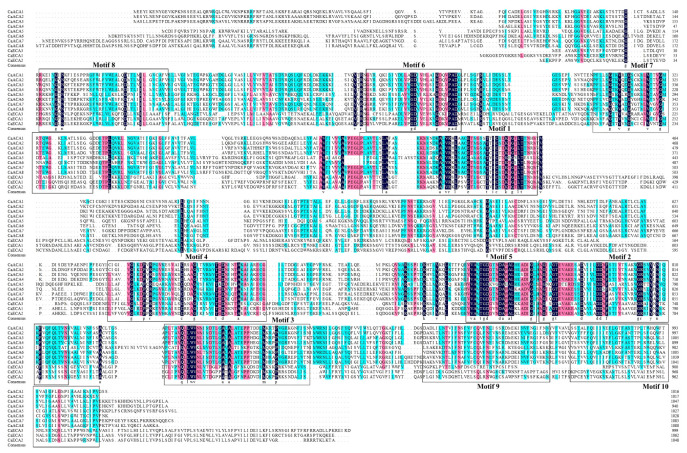
The multiple sequence alignment of CaACA/ECA proteins conducted using DNAMAN. The conserved motifs are marked by black boxes. The sequences marked in blue are conservative, pink are more conservative, and black are the most conservative.

MEME was used to analyze the ten conserved motifs in the CaACA/ECA protein sequences to gain a deeper understanding of the characteristics of the CaACA/ECA proteins. The presence of all ten motifs in each of the eleven CaACA/ECA proteins suggests a high degree of conservation throughout evolutionary history (Figure 3). The results of further analyses revealed that Motif 1 contains a continuously conserved D-K-T-G-T-L-T sequence at its end, Motif 3 contains a continuously conserved L-L-W-V-N sequence, and Motif 7 contains four discontinuous highly conserved glycine (G) residues (Figure 3 and Figure 4). The analysis of the conserved functional domains of the proteins (Figure S1) shows that the three conserved functional domains of the ACA/ECA gene family proteins correspond to three distinct conserved motifs in CaACA/ECA proteins. Specifically, the Haloacid dehalogenase-like hydrolase domain corresponds to Motif 1, the C-terminal cation-transporting ATPase domain corresponds to Motif 3, and the E1-E2 ATPase domain corresponds to Motif 7 [18].

### 2.4. Analysis of Cis-Acting Elements in the Promoter of CaACA/ECA Genes

**Figure 5:**
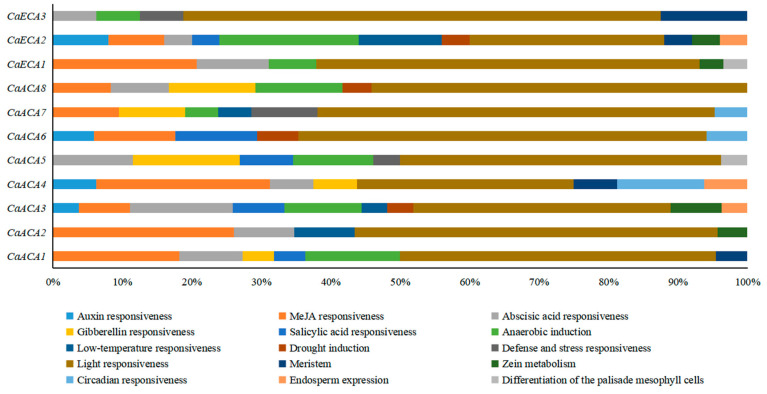
Percentage of each cis-acting element in the promoter region of the *CaACA*/*ECA* gene. Different cis-acting elements are represented by different colored rectangles.

Cis-acting elements are noncoding DNA sequences located in gene-promoter regions, which are important for gene expression and extensively participate in the regulation of various plant growth and development processes [19]. We found that the highest percentage (47.97%) of the *CaACA*/*ECA* cis-acting elements is related to the light response. Additionally, plant-hormone-related cis-acting elements, such as auxin response elements (5), methyl jasmonate response elements (30), abscisic acid response elements (19), gibberellin response elements (11), and salicylic acid response elements (8), were identified in the promoter regions of *CaACA*/*ECAs*. Plant-growth-related cis-acting elements were also identified in these promoter regions (Figure 5 and Figure S2). These results indicate that the pepper *ACA*/*ECA* gene family responds to various hormones or environmental signals.

### 2.5. Expression of CaACA/ECAs Under Various Exogenous Ca^2+^ Concentrations

**Figure 6:**
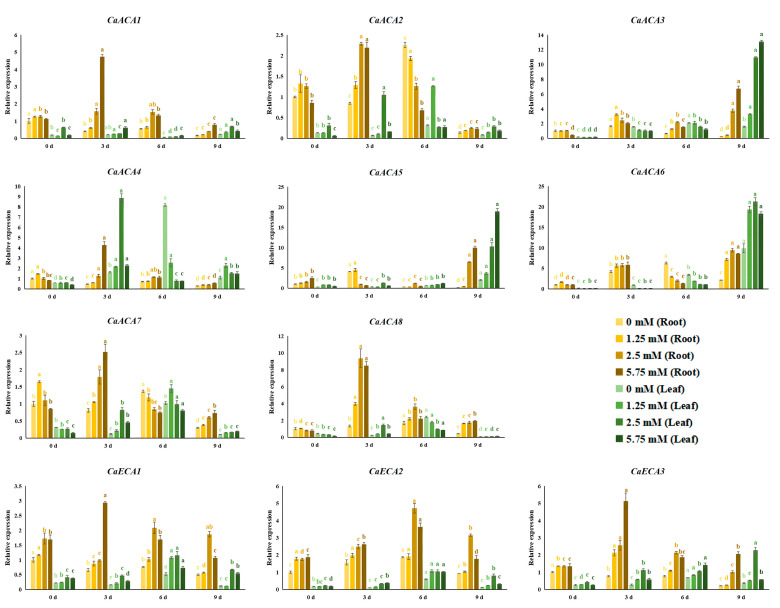
Expression dynamics of *CaACA*/*ECA* genes under various exogenous Ca^2+^ concentrations. Different letters with the same color indicate significant differences between 0, 3, 6, and 9 d (*p <* 0.05). The relative expression levels were calculated using the 2^−∆∆Ct^ method with three replicates; *β-TUB* was used as the reference gene. The root samples from 0 mM Ca^2+^ treatment on day 0 were used as the control. The values are presented as the means and standard deviations obtained from three biological replicates.

We conducted qRT-PCR analyses to investigate the expression dynamics of *CaACA*/*ECA* genes under various exogenous Ca^2+^ concentrations. The results indicate that as the treatment duration increases, the expression levels of most *CaACA*/*ECA* genes initially increase and then decrease or exhibit the opposite trend: some *CaACA*/*ECA* genes were gradually up- or downregulated. For example, *CaACA6* expression was induced by calcium deficiency (0 mM Ca^2+^), initially increasing and then decreasing in the pepper roots, reaching a peak on the sixth day, while gradually increasing in the leaves. The expressions of *CaACA3*, *CaACA4*, *CaACA5*, and *CaACA6* in the leaves were also induced by calcium deficiency, with *CaACA3* and *CaACA4* exhibiting unimodal trends, whereas *CaACA5* and *CaACA6* gradually increased. In contrast, the expression level of *CaACA5* in the roots initially decreased and then increased under 5.75 mM Ca^2+^ treatment. Moreover, the expression levels of most of the *CaACA*/*ECA* genes recovered to their original levels on the ninth day, lagging behind or exceeding the initial levels, suggesting a possible self-regulation mechanism in plants (Figure 6).

### 2.6. Expression Analysis of CaACA/ECAs Under ABA Hormone

**Figure 7:**
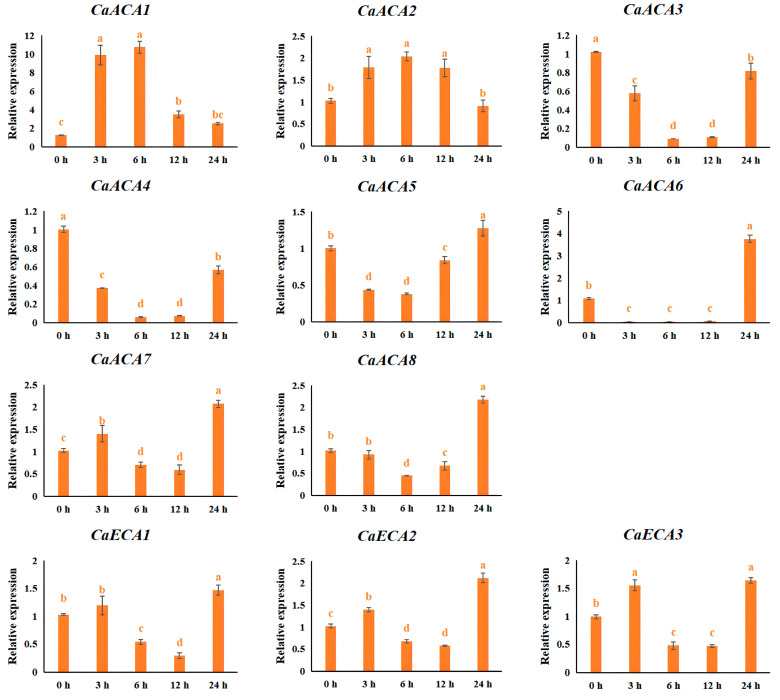
Expression dynamics of *CaACA*/*ECA* genes under ABA treatment. The relative expression levels were calculated using the 2^−∆∆Ct^ method with three replicates; *β-TUB* was used as a reference gene. The leaves prior to ABA spraying were used as the control (0 h). The values are presented as the means and standard deviations obtained from three biological replicates. Different letters indicate significant differences (*p <* 0.05).

The *ACA*/*ECA* genes of some plants respond to *ABA* [12,13,14]. We evaluated the expression dynamics under ABA treatment in peppers to investigate whether *CaACA*/*ECA*s also respond and adapt to ABA. The qRT-PCR results show that the expression levels of the eleven *CaACA*/*ECA*s exhibit different temporal patterns under ABA treatment. The expression levels of *CaACA1* and *CaACA2* were first upregulated at 3 h and were the highest 6 h after ABA treatment, indicating that *CaACA1* and *CaACA2* are the most sensitive genes in the adaptation to ABA. After ABA treatment, the expressions of *CaACA7*, *CaECA1*, *CaECA2*, and *CaECA3* were upregulated at 3 h and then downregulated, but were then rapidly upregulated again at 24 h. The expressions of *CaACA3*, *CaACA4*, *CaACA5*, *CaACA6*, and *CaACA8* were first downregulated at 3 h and were the lowest at 6 h (Figure 7), suggesting that they might also be influenced by other environmental factors (light, temperature, etc.).

### 2.7. Expression Analysis of CaACA/ECAs Under Salt Stress

**Figure 8:**
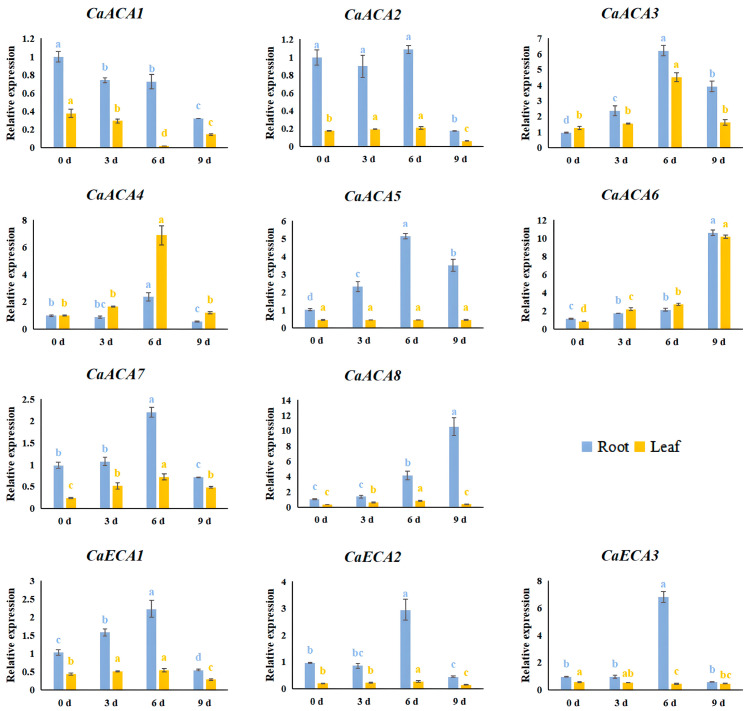
Expression dynamics of *CaACA*/*ECA* genes under salt stress. The relative expression levels were calculated using the 2^−∆∆Ct^ method from three replicates; *β-TUB* was used as the reference gene. The root samples taken before NaCl irrigation were used as the control (0 d). The values are presented as the means and standard deviations obtained from three biological replicates. Different letters within the same color indicate significant differences between 0, 3, 6, and 9 d (*p* < 0.05).

We conducted qRT-PCR analysis to evaluate the expression dynamics of the eleven identified *CaACA*/*ECA* genes to investigate their responses and adaptations to salt stress. The qRT-PCR results indicate that the expression level of *CaACA1* was suppressed under NaCl treatment. The expression patterns of *CaACA5* in the leaves did not differ over time under NaCl treatment, implying that *CaACA5* might not respond to salt stress. Other *CaACA*/*ECA* genes were induced at various times after exposure to salt stress, indicating that they are the genes involved in adaptation to salt stress (Figure 8).

## 3. Discussion

Calcium (Ca^2+^), a secondary macronutrient, acts in plant metabolism through molecular signaling [20]. Maintaining Ca^2+^ homeostasis is crucial for plants to effectively respond to various environmental stimuli. Calcium-transporting ATPase, as a vital protein for calcium transport, plays important roles in plant signaling pathways, growth and development, and abiotic stress [21]. ER-localized ACA1, ACA2, and ACA7 are crucial for plant growth and pollen fertility [22]. Inhibiting ER-type Ca^2+^-ATPases with cyclopiazonic acid severely disrupts Ca^2+^-induced stomatal closure [23,24]. Additionally, the ER-resident Ca^2+^-ATPase ECA1 is essential for maintaining the cytosolic Ca^2+^ balance and conferring tolerance to osmotic stress [25]. ACA4 and ACA11 are vital in the tonoplast for flg22-triggered Ca^2+^ signaling and related defense mechanisms [26]. The *ACA*/*ECA* gene family has been identified and functionally analyzed in various plants. However, reports on the *ACA*/*ECA* gene family in the pepper genome are lacking, and studies on the functions of this gene family are limited. Thus, investigating *ACA*/*ECA* genes in pepper is essential for advancing our understanding of Ca^2+^ regulation and stress response pathways in plants. In this study, we used various bioinformatics methods to identify the *ACA*/*ECA* gene family in pepper, analyze their expression dynamics under various exogenous Ca^2+^ concentrations, and predict their responses and adaptations to abiotic stresses.

In this study, a total of eleven *ACA*/*ECA* family members were identified in the pepper genome. Chromosome localization was analyzed for members of the *CaACA*/*ECA* gene family, and the eleven members identified were unevenly distributed on eight chromosomes. *CaACA6* and *CaECA2* are both located on chromosome 8, and *CaECA1* and *CaECA3* are both located on chromosome 11 (Figure 1A). Comparing their gene structures showed that *CaACA6* and *CaECA2*, as well as *CaECA1* and *CaECA3*, have similar intron–exon structures, which indirectly confirms that they are closely related evolutionarily. Although notable differences were found in the number of exons among the *CaACA*/*ECA* gene family members, the number of exons in the same branch is relatively conserved, and the exon/intron composition patterns are similar. In addition, the *CaACA5* gene lacks introns, whereas the other *CaACA* genes contain at least one intron, indicating that this gene may have lost all its introns during evolution (Figure 1B). The results of the physicochemical property analyses show that the CaACA/ECA proteins differ in length, isoelectric point, molecular weight, and hydrophilicity, suggesting broad functional specificity of the family (Table 1).

The results of the phylogenetic tree analysis suggest that most CaACA/ECA proteins cluster with AtACA/ECA and SlyACA/ECA proteins rather than OsACA/ECA proteins, indicating a strong evolutionary connection between pepper, Arabidopsis, and tomato. We inferred that this is because rice is a monocotyledon, whereas Arabidopsis, pepper, and tomato are dicotyledons (Figure 2A). The collinear relationships of *CaACA*/*ECA* among Arabidopsis, rice, and tomato provide insights into the functions of pepper *ACA*/*ECA* genes. The interspecific collinearity analyses revealed that *CaACA*/*ECA* exhibits seven collinear gene pairs with Arabidopsis, one collinear gene pair with rice, and twelve collinear gene pairs with tomato. Pepper and tomato have the highest number of *ACA*/*ECA* collinear gene pairs, likely due to both species being part of the Solanaceae family. In contrast, pepper and rice have only one *ACA* collinear gene pair, likely because the genetic relationship between the two species is relatively distant (Figure 2B and Table S1). In the phylogenetic tree and collinearity analysis, the collinear genes of *ACA*/*ECA* were found to be closely correlated, indicating that they perform similar functions.

Studying the cis-acting regions in gene promoters is crucial for comprehending gene regulation and forecasting gene functionalities [27]. The cis-elements identified in this study can be classified into three major categories: growth and development, the phytohormone response, and the stress response. We found that most of the *CaACA*/*ECA* cis-acting elements are related to the light response, indicating that *CaACA*/*ECA* genes participate in the complex regulatory networks in the plant response to light. Additionally, plant-hormone- and stress-related cis-acting elements were identified in these promoter regions, accounting for 29.67% and 14.63% of the total, respectively (Figure 5 and Figure S2). Collectively, these results demonstrate that *CaACA*/*ECA* gene family members might be transcriptionally regulated by a wide range of developmental processes, multiple hormones, and various stresses, and these data will facilitate the understanding of the regulatory networks of *CaACA*/*ECA* genes in various biological processes.

Calcium promotes plant growth and development, as well as enhances fruit yield and quality [3]. Calcium nutrition positively affects pepper growth, photosynthesis, yield, and quality [28,29]. Ca^2+^ absorption by plant cells involves two processes: uptake and efflux. However, cells cannot indefinitely absorb Ca^2+^, as an excessively high intracellular Ca^2+^ concentration can be toxic. Hence, the absorption of Ca^2+^ by cells also maintains the intracellular Ca^2+^ balance. This balance is maintained by Ca^2+^ transporters, specifically ACA/ECAs, which are responsible for either expelling Ca^2+^ from the cells or pumping it into organelles, such as the endoplasmic reticulum and vacuoles, for storage. This ensures that the intracellular free Ca^2+^ concentration remains low [30]. The expression levels of the majority of *ACA* genes in bananas were high when subjected to 2 and 4 mM Ca^2+^ treatments, whereas their expressions were substantially suppressed under 0 and 1 mM Ca^2+^ treatments [16]. Bananas might expel Ca^2+^ from the cells or pump Ca^2+^ into organelles by upregulating *ACA* expression. Arabidopsis, an *ateca1-1* mutant, displayed Ca^2+^ deficiency symptoms under 0.2 to 0.4 mM Ca^2+^ conditions [31]. In this study, the qRT-PCR results show that the expression level patterns of eleven *CaACA*/*ECA*s temporally differed under various exogenous Ca^2+^ concentrations, suggesting that CaACA/ECAs are sensitive to variations in Ca^2+^ concentration. Under 1.25, 2.5, and 5.75 mM Ca^2+^ treatments, the expression levels of most of the *CaACA*/*ECA* genes peaked on the third or sixth day, whereas some *CaACA*/*ECA* genes (*CaACA3*, *CaACA5*, and *CaACA6*) were rapidly upregulated on the ninth day (Figure 6). We speculated that the peppers maintained their intracellular Ca^2+^ balance by upregulating the expression levels of these genes, and Ca^2+^ was expelled from the cells or pumped into the endoplasmic reticulum or vacuoles for storage. Further research is needed to confirm this hypothesis.

Exogenous ABA can induce the expressions of *ACA*/*ECA* genes to a certain extent. The exogenous application of ABA during the Arabidopsis seedling stage markedly increased the expression levels of *AtACA8*, *AtACA9,* and *AtACA10* [12,32]. ABA stress in rice strongly activates the expression of *OsACA6* [13]. In this study, the expression levels of most of the *CaACA*/*ECA* genes were upregulated with different temporal expression patterns after ABA treatment, indicating that *CaACA*/*ECA* genes may be involved in the ABA-mediated stress response (Figure 7).

Salt stress inhibits the growth of peppers. For example, 0.2 M NaCl strongly inhibited the germination of pepper seeds and the growth of seedlings [33]. However, the expressions of *ACA*/*ECA* genes can be induced by salt stress for adaptations or as part of an adaptation mechanism. The heterologous expression of *AtACA2* in yeast strain K616 enhanced its survival rate under 300 mM NaCl stress, indicating that *AtACA2* is a salt-tolerance gene [34]. *OsACA5* and *OsACA6* expression levels substantially increased under salt stress in rice [14]. The expression levels of *SlyECA1* notably increased under 50 mM NaCl treatment in tomato [35]. In this study, the qRT-PCR results indicated that most of the *CaACA*/*ECA* genes were induced at various times after NaCl stress, indicating that they respond or adapt to salt stress (Figure 8). In addition, CaACA1 and AtACA2 are in the same evolutionary branch; however, the expression level of *CaACA1* was suppressed under NaCl treatment, which requires further research to understand.

## 4. Materials and Methods

### 4.1. Identification of ACA/ECA Family Members in Pepper

The ACA/ECA family protein sequences were downloaded from the Arabidopsis database (https://www.Arabidopsis.org/, accessed on 19 August 2024) and then aligned using the BlastP function from NCBI (https://blast.ncbi.nlm.nih.gov/Blast.cgi#, accessed on 19 August 2024) within the pepper genome database to identify candidate genes (with parameters E < 1.0 × 10^−10^ and identity > 40%). Subsequently, to eliminate the sequences lacking conserved domains, the online resources CDD (https://www.ncbi.nlm.nih.gov/cdd, accessed on 19 August 2024) [36] and PFAM (http://pfam.xfam.org, accessed on 19 August 2024) [37] were used to analyze the candidate gene sequences. After the above screening process, the genes that remained were members of the pepper *ACA*/*ECA* family. Additionally, protein sequences were analyzed using the online platform ExPASy (http://web.expasy.org/protparam/, accessed on 19 August 2024) to predict the amino acid length, molecular weight, theoretical isoelectric point (pI), overall average hydrophilicity, and other related parameters [38]. Cell-PLoc 2.0 (http://www.csbio.sjtu.edu.cn/bioinf/Cell-PLoc-2/, accessed on 19 August 2024) was used for the subcellular localization prediction of protein sequences [39].

### 4.2. Chromosomal Location and Gene Structure Analysis

The position of each *ACA*/*ECA* gene on the twelve pepper chromosomes was determined from the pepper genome database, and a genetic linkage map was constructed using TBtools [40].

The gene structures of the *ACA*/*ECA* family genome sequence and the CDS sequence of pepper were analyzed using online software GSDS 2.0 (http://gsds.cbi.pku.edu.cn, accessed on 20 August 2024) [41].

### 4.3. Protein Phylogenetic and Gene Collinearity Analyses of ACA/ECA in Arabidopsis, Rice, Tomato, and Pepper

The rice and tomato ACA/ECA family protein sequences were downloaded from NCBI (https://www.ncbi.nlm.nih.gov/, accessed on 20 August 2024). MEGA11 software was used to construct a phylogenetic tree and perform multiple sequence alignment between Arabidopsis, rice, tomato, and pepper ACA/ECA proteins, with 1000 bootstrap replications [42].

The gene annotation files (GFFs) and genome files (FASTAs) for Arabidopsis, rice, tomato, pepper were downloaded from NCBI. Then, TBtools-II software was used to visualize and analyze the collinearity results.

### 4.4. Conserved Motif Analysis

MEME (http://meme-suite.org/, accessed on 21 August 2024) was used to analyze the conserved motifs of the ACA/ECA family protein sequence in pepper, with a motif count of ten [43]. Finally, TBtools software was used for visual mapping. DNAMAN was used for pepper ACA/ECA protein sequence homology alignment.

### 4.5. Cis-Acting Elements Analysis

The upstream sequence (2 kb) of the start codon of each gene was extracted to determine the number of cis-elements in the pepper *ACA*/*ECA* gene promoter. The cis-elements of these gene promoter regions were analyzed using PlantCARE software (http://bioinformatics.psb.ugent.be/webtools/plantcare/html/, accessed on 21 August 2024) [44]. EXCEL 2016 was used to visualize the results.

### 4.6. Plant Material and Treatments

Experiments were conducted from July to September 2024 at the Vegetable Research Institute of Beijing Academy of Agricultural and Forestry Sciences (116°29′ E, 39°94′ N) in a solar greenhouse. Pepper cultivar JINGXUAN NO.103 was used as the experimental material. Pepper seeds were sown in a growing medium (peat/vermiculite/perlite = 3:1:1). Following 40 d of growth, the seedlings were subjected to various Ca^2+^ concentrations (0, 1.25, 2.5, and 5.75 mM) and abiotic stress (100 μM ABA and 0.2 M NaCl) treatments [45].

The seedlings were treated with Hoagland nutrient solution, adjusted to the aforementioned Ca^2+^ concentrations to investigate the expression dynamics of the *ACA*/*ECA* family genes under various exogenous Ca^2+^ concentrations (the Ca^2+^ concentration was adjusted using Ca(NO_3_)_2_, and the other components were consistent with the Hoagland nutrient solution formula). Leaf and root samples were collected at 0, 3, 6, and 9 d after treatment. The root samples from the 0 mM Ca^2+^ treatment at 0 d were used as the control. For the ABA treatment, leaf samples were collected at 3, 6, 12, and 24 h after spraying ABA on the leaves. The leaves before ABA spraying were used as the control (0 h). For the NaCl treatment, the seedlings were irrigated with Hoagland nutrient solution containing 0.2 M NaCl. Then, the samples (leaves and roots) were collected at 3, 6, and 9 d after treatment. The root samples taken before NaCl irrigation were used as the control (0 d). Each sample was collected in triplicate as biological replicates. Upon collection, every sample was promptly frozen in liquid nitrogen and kept in an ultra-low-temperature refrigerator at −80 °C for storage until RNA extraction. The components of the Hoagland nutrient solution are listed in Table S2.

### 4.7. RNA Extraction, cDNA Synthesis, and Quantitative Real-Time PCR

The total RNA was extracted from the plant material using a FastPure Universal Plant Total RNA Isolation Kit (Vazyme, Nanjing, China). Then, 1 μg of total RNA was reverse transcribed to synthesize cDNA using a HiScript III 1st Strand cDNA Synthesis Kit (+gDNA wiper) (Vazyme, Nanjing, China). qRT-PCR assays were performed using a Bio-Rad CFX Opus 96 real-time PCR system (Bio-Rad, Hercules, CA, USA) to acquire the cycle threshold (C_t_). The reaction system comprised the following components: SYBR Green Mix (2×) 10.0 μL (TOYOBO, Osaka, Osaka Prefecture, Japan), upstream and downstream primers (0.5 μL each), and 2.0 μL of cDNA, with the volume adjusted to 20.0 μL using ddH_2_O. For the experiment, three biological replicates were established, and *β-TUB* was used as the reference gene. The 2^−∆∆Ct^ method was used to calculate the relative gene expression levels [46]. The primers used for qRT-PCR are listed in Table S3.

### 4.8. Statistical Data Analysis

Multiple comparisons were conducted using PASW Statistics 18 (LSD and Waller–Duncan methods) to test the significance of the differences in the data. EXCEL 2016 was used to analyze the experimental data and generate charts. Different letters in the figures indicate significant differences between the treatments (*p* < 0.05).

## 5. Conclusions

In this study, eleven *ACA*/*ECA* pepper genes were identified and characterized. The distribution of these eleven *ACA*/*ECA* genes is disproportionate across the eight pepper chromosomes. The gene structures in the same evolutionary branch are relatively conserved. The genetic relationship of *ACA*/*ECA* genes between pepper and dicotyledonous plants is relatively close. The presence of all ten motifs in each of the eleven CaACA/ECA proteins suggests a high degree of conservation throughout evolutionary history. *CaACA*/*ECA* contains various plant-hormone-, growth-, and stress-related cis-acting elements. The qRT-PCR results indicate that *CaACA*/*ECA*s are sensitive to variations in Ca^2+^ concentrations. Most *CaACA*/*ECA* genes were induced at various times after ABA treatment and NaCl stress, indicating that they respond or adapt to ABA and salt stress. This study established a theoretical foundation for studying the functions and mechanisms of pepper *ACA*/*ECA* genes in plant development and their responses to abiotic stresses.

## Figures and Tables

**Table 1:** Detailed information on calcium-transporting ATPase of pepper (CaACA/ECAs).

Gene ID	Gene Name	No. of Amino Acids	Molecular Weight (Da)	pI	Instability Index	Grand Average Hydropathicity	Chr	Subcellular Localization
LOC107867231	CaACA1	1016	111,116.61	5.69	36.61	0.168	4	Chloroplast, Endoplasmic reticulum, Vacuole
LOC107858641	CaACA2	1017	111,284.94	5.73	36.09	0.173	2	Chloroplast, Endoplasmic reticulum, Vacuole
LOC107870028	CaACA3	1047	114,255.38	6.38	37.4	0.112	5	Chloroplast, Endoplasmic reticulum, Vacuole
LOC107843905	CaACA4	940	103,249.83	5.9	33.84	0.303	10	Chloroplast, Endoplasmic reticulum, Vacuole
LOC107865779	CaACA5	1027	113,519.95	8.96	34.16	0.08	3	Cell membrane
LOC107879281	CaACA6	1028	114,121.77	8.69	33.07	0.023	8	Cell membrane
LOC107877781	CaACA7	1083	117,817.43	7.24	38.9	0.09	7	Cell membrane
LOC107861599	CaACA8	1080	117,887.24	6.63	37.59	0.095	3	Cell membrane
LOC107847413	CaECA1	1062	116,058.54	5.23	39.56	0.042	11	Endoplasmic reticulum
LOC107839342	CaECA2	1048	115,947.41	5.65	38.06	0.077	8	Endoplasmic reticulum
LOC107847547	CaECA3	1000	109,560.28	5.8	38.88	0.252	11	Cell membrane
	Average	1031.64	113,165.40	6.54	36.74	0.129		

## Data Availability

All the other data sets supporting the conclusions of this article are included within the article and its Supplementary Materials.

## Supplementary Materials

The following supporting information can be downloaded at: https://www.mdpi.com/article/10.3390/ijms252312822/s1.
