# Supplementary material for: Genome-Wide Identification and Expression Analysis of ACA/ECAs in Capsicum annuum L
Source: Int J Mol Sci. 2024 Nov 28;25(23):12822. doi: 10.3390/ijms252312822 (PMC11641697; doi:10.3390/ijms252312822)
Supplement: Supplementary file 1 [file ijms-25-12822-s001.zip › ijms-3311578-supplementary.pdf]

Supplementary Figures and Tables

Table S1. Collinearity relationships of pepper *ACA/ECA* genes with Arabidopsis, rice and tomato.

Chromosome of other species	The corresponding *ACA/ECA* gene	Collinearity	Chromosome of pepper	The corresponding *CaACA/ECA* gene
At-1	*AtACA1*	==	Ca-4	*CaACA1*
At-2	*AtACA7*	==	Ca-2	*CaACA2*
At-2	*AtACA4*	==	Ca-5	*CaACA3*
At-3	*AtACA11*	==	Ca-5	*CaACA3*
At-2	*AtACA4*	==	Ca-10	*CaACA4*
At-3	*AtACA11*	==	Ca-10	*CaACA4*
At-3	*AtACA13*	==	Ca-3	*CaACA5*
Os-3	*OsACA3*	==	Ca-4	*CaACA1*
Sly-2	*SlyACA2*	==	Ca-4	*CaACA1*
Sly-4	*SlyACA1*	==	Ca-4	*CaACA1*
Sly-2	*SlyACA7*	==	Ca-2	*CaACA2*
Sly-2	*SlyACA2*	==	Ca-2	*CaACA2*
Sly-10	*SlyACA4*	==	Ca-5	*CaACA3*
Sly-4	*SlyACA3*	==	Ca-5	*CaACA3*
Sly-10	*SlyACA4*	==	Ca-10	*CaACA4*
Sly-4	*SlyACA3*	==	Ca-10	*CaACA4*
Sly-9	*SlyACA5*	==	Ca-3	*CaACA5*
Sly-7	*SlyACA11*	==	Ca-7	*CaACA7*
Sly-3	*SlyACA8*	==	Ca-3	*CaACA8*
Sly-1	*SlyECA2*	==	Ca-8	*CaECA2*

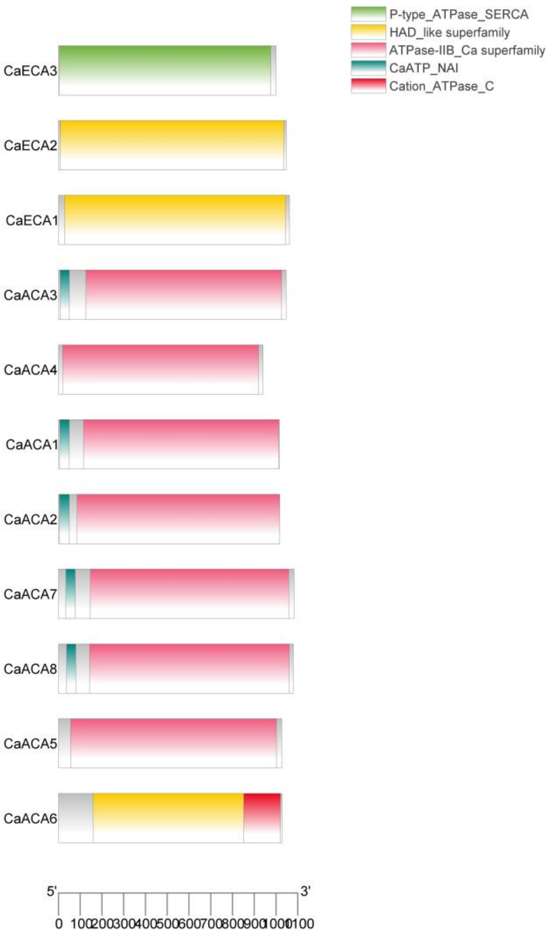

Figure S1. Domain architecture of CaACA/ECA proteins.

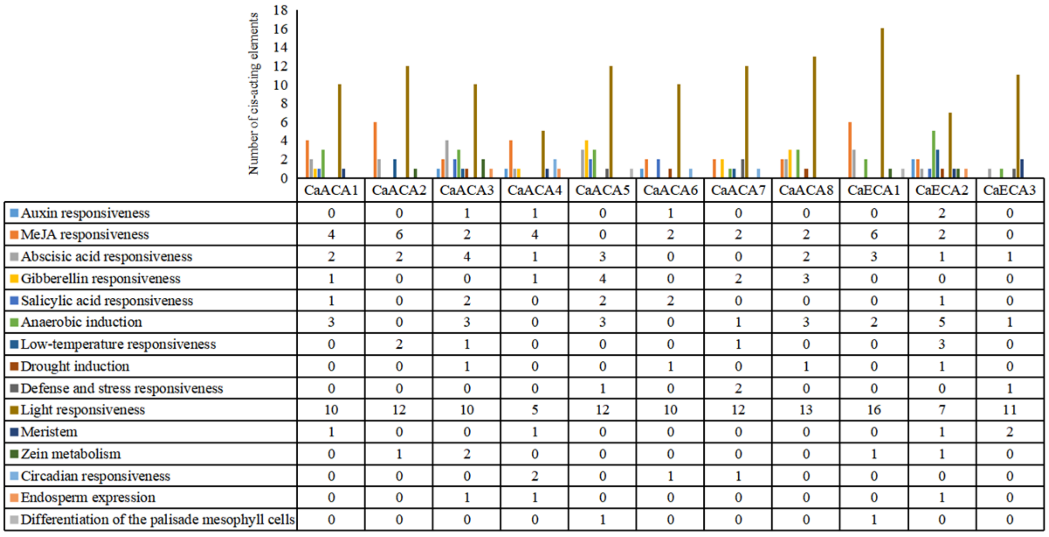

	CaACA1	CaACA2	CaACA3	CaACA4	CaACA5	CaACA6	CaACA7	CaACA8	CaECA1	CaECA2	CaECA3
Auxin responsiveness	0	0	1	1	0	1	0	0	0	2	0
MeJA responsiveness	4	6	2	4	0	2	2	2	6	2	0
Abscisic acid responsiveness	2	2	4	1	3	0	0	2	3	1	1
Gibberellin responsiveness	1	0	0	1	4	0	2	3	0	0	0
Salicylic acid responsiveness	1	0	2	0	2	2	0	0	0	1	0
Anaerobic induction	3	0	3	0	3	0	1	3	2	5	1
Low-temperature responsiveness	0	2	1	0	0	0	1	0	0	3	0
Drought induction	0	0	1	0	0	1	0	1	0	1	0
Defense and stress responsiveness	0	0	0	0	1	0	2	0	0	0	1
Light responsiveness	10	12	10	5	12	10	12	13	16	7	11
Meristem	1	0	0	1	0	0	0	0	0	1	2
Zein metabolism	0	1	2	0	0	0	0	0	1	1	0
Circadian responsiveness	0	0	0	2	0	1	1	0	0	0	0
Endosperm expression	0	0	1	1	0	0	0	0	0	1	0
Differentiation of the palisade mesophyll cells	0	0	0	0	1	0	0	0	1	0	0

Figure S2. The number of cis-acting elements of *CaACA/ECA* genes.

Table S2. The components of Hoagland nutrient solution.

Nutrient solution composition	Concentration (mg/L)
$Ca(NO_3)_2$	945
K_2SO_4	607
$NH_4H_2PO_4$	115
$MgSO_4$	493
EDTA ferric-sodium salt	20
$FeSO_4$	15
H_3BO_3	2.86
$Na_2B_4O_7 \cdot 10H_2O$	4.5
$MnSO_4$	2.13
$CuSO_4$	0.05
$ZnSO_4$	0.22
$(NH_4)_2SO_4$	0.02

Note: Hoagland nutrient solution was purchased from Qingdao Hope Bio-Technology Co. , Ltd.

Table S3. The qRT-PCR primers used in this study.

Gene name	Primer sequence (5'-3')
β-TUB	F: GAGGGTGAGTGAGCAGTTC<br>R: CTTCATCGTCATCTGCTGTC
CaACA1	F: CCTGGGAACACCGACTGAAA<br>R: TTACAGTGTGCACGGAGACC
CaACA2	F: TGTTCGGATGGTCACTGGAG<br>R: GTGTGCTTGTCTAGCGGAGA
CaACA3	F: ACCACATGCAATTTCCAGGAT<br>R: GTCTTTTGCCAGTTCAGCCC
CaACA4	F: TCTGCATGTGAGACGATGGG<br>R: CAAAGCTATTGCATCCCCGC
CaACA5	F: TTAAAGCTAGCGAGCCCGAG<br>R: GGTCGATTGGTGGTTGTTGC
CaACA6	F: ACGACAGGCCAAAAACCAAG<br>R: GGACAAGAGCAACTGCAACG
CaACA7	F: CGAGCTCCTGTTGGTCGTAG<br>R: ACAGCCCTCTCATTCGTCTC
CaACA8	F: TGCAGTTAGACAGCGGAGTG<br>R: GCAATCGCAACACACCTCAA
CaECA1	F: CGATTACTTCGTGGAGGGCA<br>R: CGACATGGCCAAAAGAAGCC
CaECA2	F: CTCCTGGGTTTTCTTCCGCT<br>R: CATTCACCCCAGTTGCGAAG
CaECA3	F: ATGGGTCAACAGTCGCTCTC<br>R: CGCACCAATCTTTTGGCACA
